# Supplementary material for: Peptide Self-Assembly Facilitating DNA Transfection and the Application in Inhibiting Cancer Cells
Source: Molecules. 2024 Feb 21;29(5):932. doi: 10.3390/molecules29050932 (PMC10934058; doi:10.3390/molecules29050932)
Supplement: Supplementary file 1 [file molecules-29-00932-s001.zip › molecules-2826573-supplementary.pdf]

Communication

Peptide Self-Assembly Facilitating DNA Transfection and the Application in Inhibiting Cancer Cells

Jingyu Wang [1,*], Min Ye [2] and Baokuan Zhu [1]

[1] School of Biomedical Engineering and Technology, Tianjin Medical University, Tianjin 300070, China; 18504891222@163.com
[2] College of Pharmacy, Southern Medical University, Guangzhou 510280, China; yemin@cuhk.edu.cn
* Correspondence: wangjy_026@tmu.edu.cn

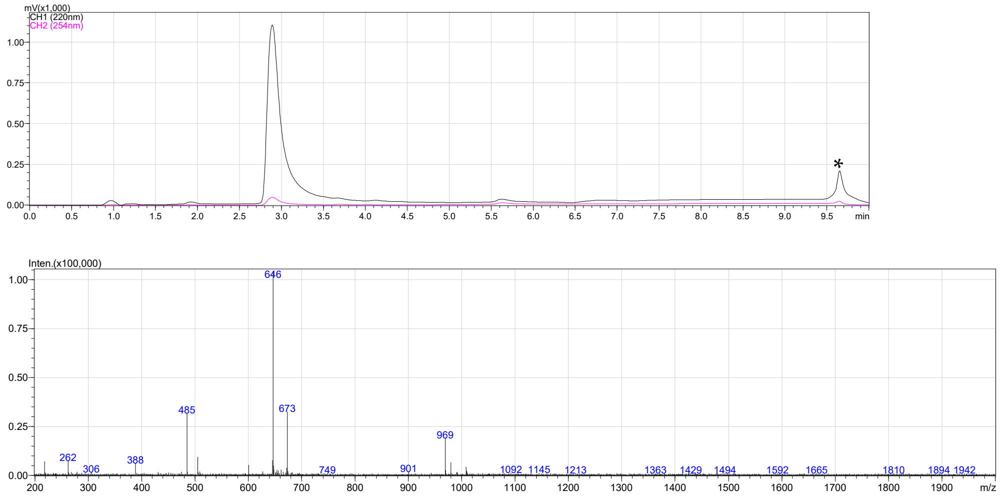

Figure S1. LC–MS of compound **1**. Calc. M = 1936.1, obsvd (1/2M + H) = 969, obsvd (1/3M + H) = 646, obsvd (1/4M + H) = 485. *: system peak.

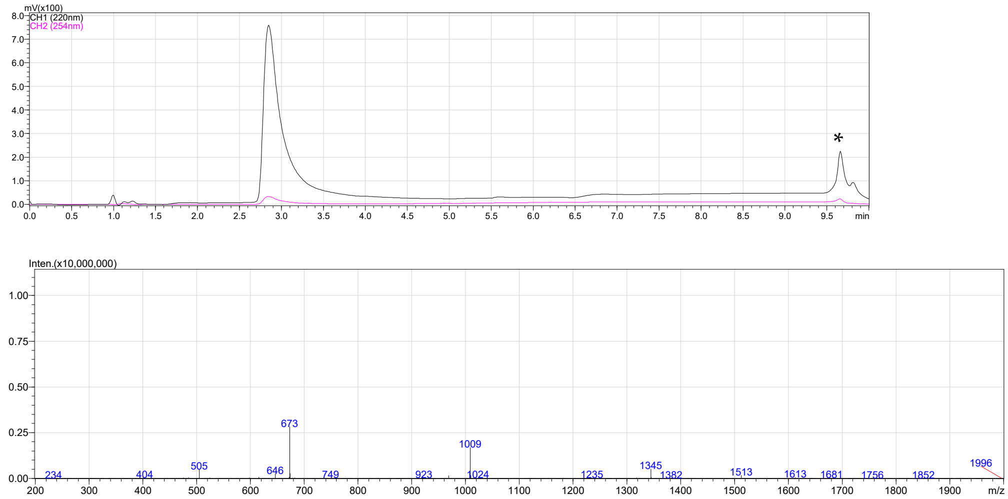

Figure S2. LC–MS of compound **2**. Calc. M = 2016.08, obsvd (1/2M + H) = 1009, obsvd (1/3M + H) = 673. *: system peak.

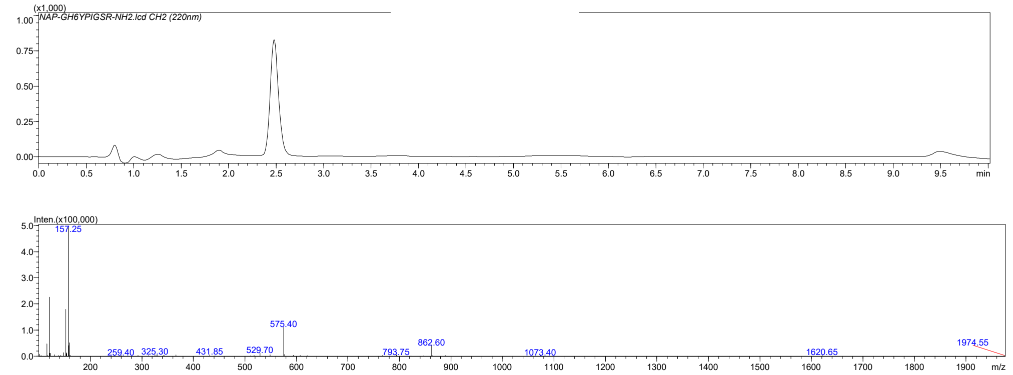

Figure S3. LC–MS of compound **3**. Calc. M = 1722, obsvd (1/2M + H) = 862.60, obsvd (1/3M + H) = 575.40.

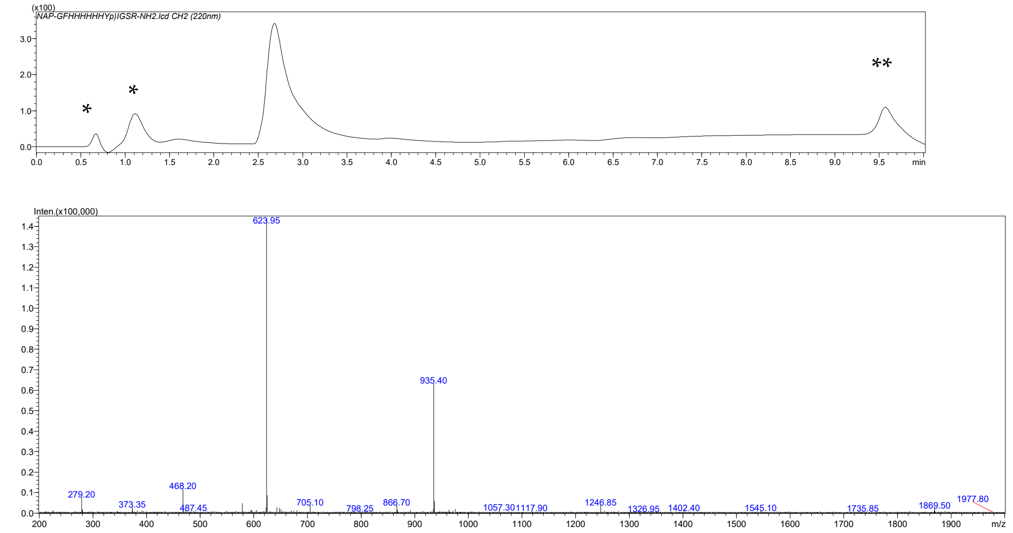

Figure S4. LC–MS of compound **4**. Calc. M = 1869, obsvd (1/2M + H) = 935.40, obsvd (1/3M + H) = 623.95, obsvd (1/4M + H) = 468.20. *: solvent peak **: system peak.

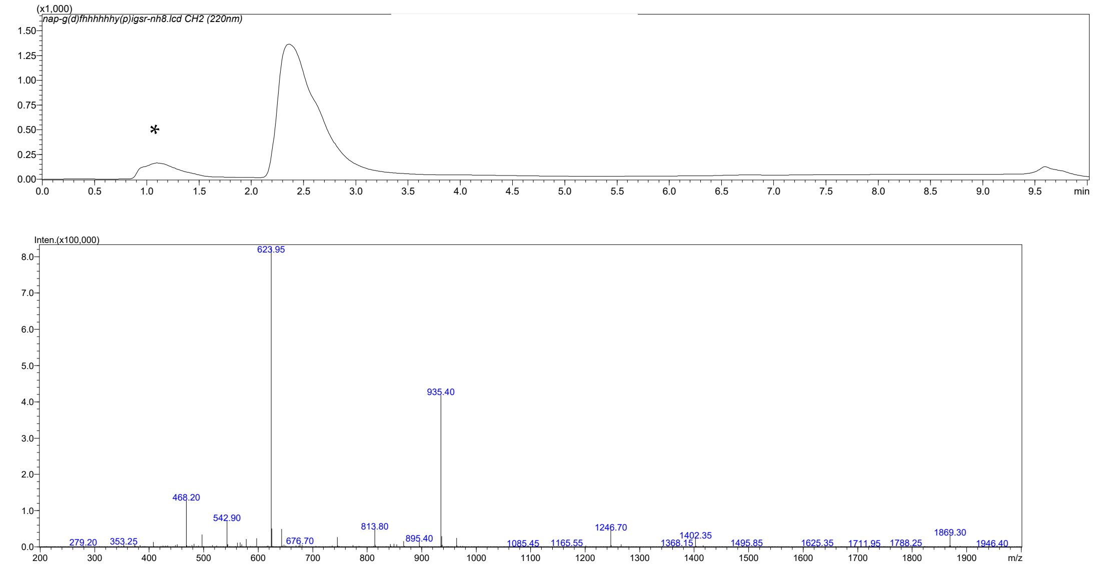

Figure S5. LC–MS of compound **5**. Calc. M = 1869, obsvd (1/2M + H) = 935.40, obsvd (1/3M + H) = 623.95, obsvd (1/4M + H) = 468.20. *: solvent peak.

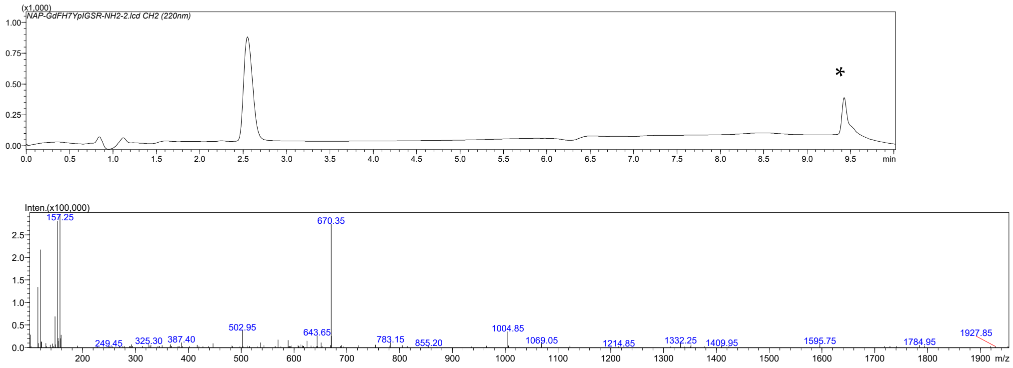

Figure S6. LC–MS of compound **6**. Calc. M = 2006, obsvd (1/2M + H) = 1004.85, obsvd (1/3M + H) = 670.35, obsvd (1/4M + H) = 502.95. *: system peak.

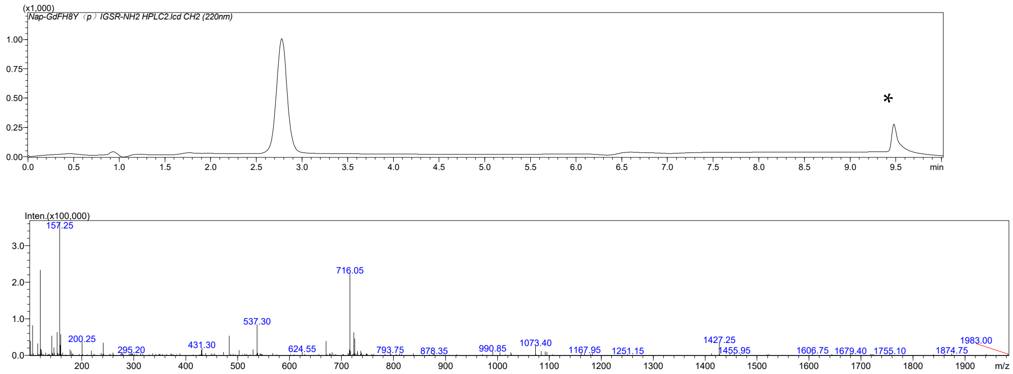

Figure S7. LC–MS of compound **7**. Calc. M = 2143, obsvd (1/2M + H) = 1073.40, obsvd (1/3M + H) = 716.05, obsvd (1/4M + H) = 537.30. *: system peak.

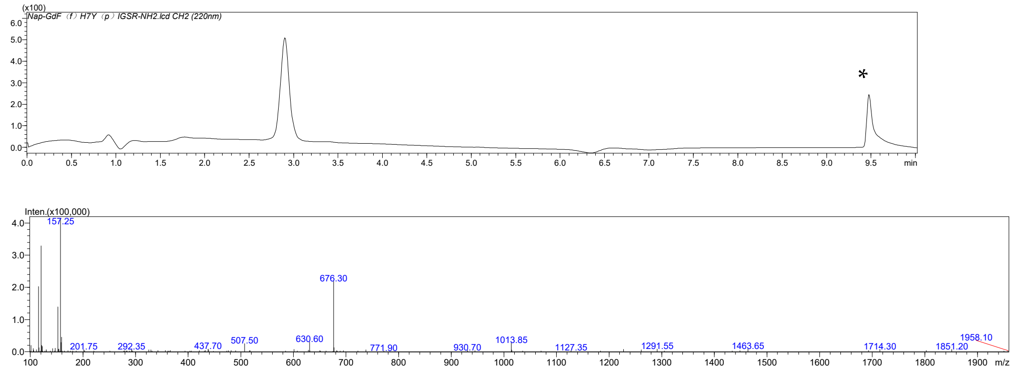

Figure S8. LC–MS of compound **8**. Calc. M = 2025, obsvd (1/2M + H) = 1013.85, obsvd (1/3M + H) = 676.30. *: system peak.

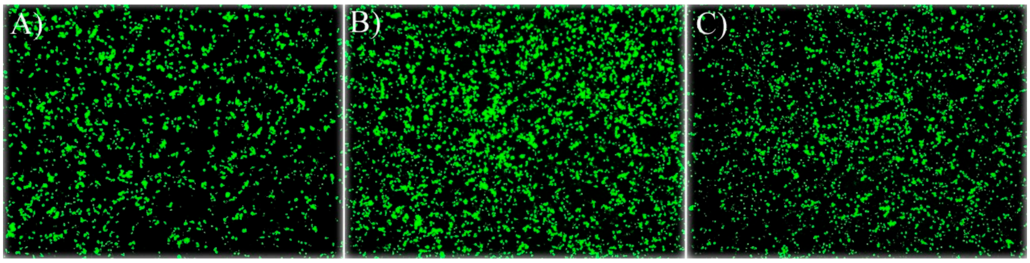

Figure S9. Fluorescent images of transfection using different vectors: (**A**) 0.75 mg/mL of compound **3** (without phenylalanine), (**B**) 0.375 mg/mL of compound **4** (one phenylalanine), (**C**) 0.375 mg/mL of compound **2** (two phenylalanines).

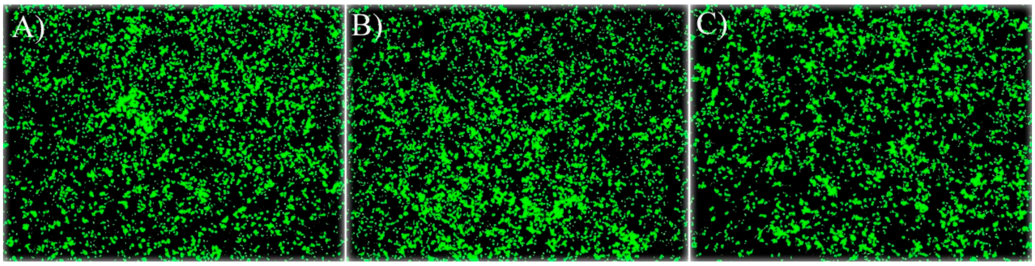

Figure S10. Fluorescent images of transfection using different vectors: (**A**) 0.375 mg/mL of compound **5** (six histidines), (**B**) 0.375 mg/mL of compound **6** (seven histidines), (**C**) 0.375 mg/mL of compound **7** (eight histidines).
